# A novel cryptic splice donor due to synonymous variant in *VPS13A* as an underlying cause of a chorea-acanthocytosis in a large family

**DOI:** 10.1016/j.heliyon.2024.e39128

**Published:** 2024-10-09

**Authors:** Majed Alluqmani, Shahid Iqbal, Sulman Basit

**Affiliations:** a Department of Neurology, College of Medicine, Taibah University Madinah, Saudi Arabia; b Shaheed Zulfiqar Ali Bhutto Medical University Islamabad, Pakistan; c Department of Biochemistry and Molecular Medicine, College of Medicine, Taibah University Madinah, Saudi Arabia; d Center for Genetics and Inherited Diseases, Taibah University Madinah, Saudi Arabia

**Keywords:** Huntington's disease, Gene mutation, Genetic diagnosis, Cryptic splice site

## Abstract

Chorea-acanthocytosis (ChAc) is a rare inherited disease of the nervous system. In this disease the neurological manifestations are associated with acanthocytosis of the red blood cells. The clinical features appear in the third to fourth decades of life. Generalized weakness, choreiform movement disorder, decline in cognition, and psychiatric symptoms are the characteristic features of the disease. The differential diagnosis between Huntington's disease and ChAc is difficult because both the diseases share similar neurological features. Herein, we recruited a large family with multiple individuals initially diagnosed as having Huntington's disease. Analysis of the DNA samples of affected individuals by exome sequencing detected a synonymous variant (NM_001018037.2; c.5040C > T) in the *VPS13A*. Multiple splice site detection tools were used to predict the potential pathogenicity of the novel synonymous variant. The variant, identified in this study, was predicted to be a cryptic splice donor site that may lead to aberrant pre-mRNA splicing. Reverse transcriptase PCR analyses of patient blood-derived RNA showed activation of a cryptic mid‐exon splice donor, leading to frameshift. The variant was confirmed in all other affected and unaffected individuals using Sanger sequencing.

This is the first report of synonymous variants of *VPS13A* as an underlying cause of ChAc. Our results provide the first direct evidence of the involvement of a synonymous variant of *VPS13A* in ChAc. Additionally, this study emphasized the importance of considering *VPS13A* gene mutations in the screening of Huntington's patients.

## 1 Introduction

Chorea-acanthocytosis (ChAc) is an inherited neuromuscular disorder. Mutations in the vacuolar protein sorting protein 13A (VPS13A) encoding gene is the underlying cause of the disease [1,2]. It is a late-onset disease, and its clinical features appear at an average age of approximately 35 years [3]. Clinical features observed in CHAC are progressive chorea, orofacial lingual dyskinesia, cognitive impairment, seizures, psychiatric symptoms, and neuromuscular manifestations with acanthocytes in peripheral blood [2,[4], [5], [6]]. Neuroimaging of the affected brain reveals atrophy of the caudate nucleus [7].

Specific clinical features and irregularly shaped red blood cells (RBCs) with spikes on the outside (acanthocytes) in the peripheral blood help in the diagnosis of CHAC. However, neuroimaging and genetic testing are very important for the exact diagnosis of CHAC due to overlapping clinical features in CHAC and other syndromes, including McLeod syndrome (MLS), pantothenate kinase-associated neurodegeneration (PKAN), Huntington's disease-like 2 (HDL2), other forms of inherited chorea (such as Huntington's disease), other forms of Huntington's-like disorders, and other syndromes of neurodegeneration with brain iron accumulation (NBIAs). CHAC is clinically difficult to distinguish from Huntington's disease (HD) because these disorders have similar symptoms and imaging findings [8]. Single-case voxel-based morphometry (VBM) and analysis of brain sections are required to differentiate CHAC and HD [8,9]. No specific treatment exists for CHAC; however, deep brain stimulation and some drugs are used to relieve the symptoms.

CHAC is a very rare disease and the incidence is very low. Our present understanding of the disease is based on a few case reports [6,10]. Here, we present the clinical manifestations and genetic findings of CHAC in five additional cases from a single family. Moreover, for the first time, we showed that a homozygous synonymous variant in *VPS13A* could be an underlying cause of the CHAC phenotype.

## 2 Methods

### 2.1 Clinical specimens

**Fig. 1:**
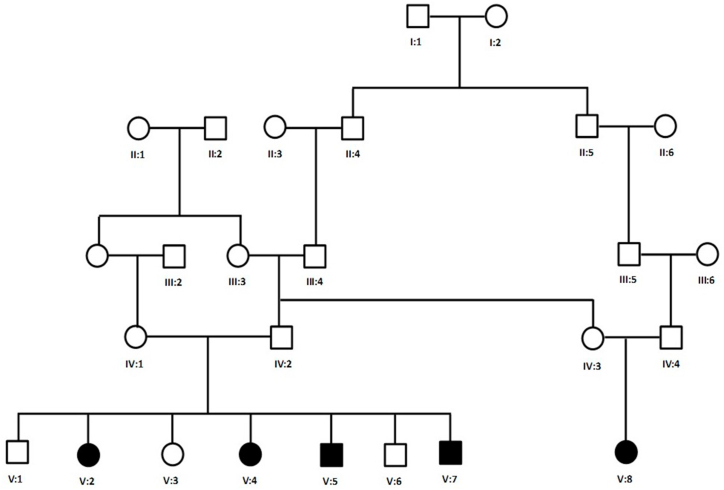
Pedigree of a family segregating autosomal recessive chorea-acanthocytosis.

In this study, we investigated a large consanguineous five-generation family with five affected individuals: three females (V:2, V:4, and V:8) and two males (V:5 and V:7) (Fig. 1). The Institutional Review Board (IRB) of Taibah University, Medina, provided ethical approval for this study. Written consents were taken from all participants for the genetic assessment and publication of the data. Three ml of peripheral blood was drawn and collected in EDTA tubes. Blood samples were collected in the Saudi-German hospital from all affected individuals (V:2, V:4, V:5, V:7, V:8), both parents (IV:1, IV:2), and three unaffected (V:1, V:3, V:6) individuals in EDTA vacutainers. Total DNA from white blood cells was isolated using a Qiaquick DNA Mini Kit (Qiagen, Germany). Genomic DNA was quantified using a Nanodrop-1000 spectrophotometer (Titertek Berthold, Germany).

### 2.2 Diagnostic tests

Clinical biochemistry, urine analysis, immunology and serology, MRI and MRA of the brain, and the Montreal Cognitive Assessment were carried out for all affected individuals.

### 2.3 Sequencing of the complete coding part of the human genome

Genomic DNA samples from two affected individuals (V:4 and V:5) were sequenced to determine the genetic changes in the complete part of the human genome. DNA libraries were pooled and sequenced using the SureSelect v6 capture kit on an Illumina NextSeq500 platform with 80 × average on-target coverage.

### 2.4 Whole exome sequence reads alignment and variant identification

The Burrows-Wheeler Aligner software package [11] was used for alignment of the raw reads with the reference human genome assembly (GRCh38/Hg38). Picard and Genome Analysis Toolkit (GATK) (https://software.broadinstitute.org/gatk/) were used for the realignment of indels, exclusion of duplicates, recalibration of quality, variant calling, and detection. ANNOVAR was used to annotate the datasets [12].

### 2.5 Variant screening and selection criteria

Intergenic variants, variants upstream and downstream of the coding regions, variants within the noncoding introns, and variants with minor allele frequency (MAF >1 %) in the 1000 genome, ExAC, and gnomAD databases were excluded from the further analysis. The variant selection criteria used in the study included CADD-phred score of more than 13, minor allele frequency of less than 0.01, present near to the splice sites (±12bp), and variants in the coding exons. *In silico* programs including REVEL [13], ClinPred [14], SIFT [15], Polyphen2 [16], LRT [17], MutationAssessor [18], PROVEAN [19], CADD [20], MutationTaster [21], dbscsnv11_AdaBoost [22], dbscsnv11_RandomForest [22], and Human Splicing Finder (HSF) [23] have been used to calculate the deleterious effect of the variant on the structure and function of the proteins. Genotype-phenotype analyses were performed using the Exomiser [24] and Phenolyzer [25] tools. Finally, variants were categorized according to the standards and guidelines of the American College of Medical Genetics and Genomics (ACMG) [26].

### 2.6 Reverse transcription of total RNA and sequencing of cDNA

The effect of the synonymous variant of the *VPS13A* gene in the proband was validated using reverse transcription of the total RNA of the proband. PCR amplification and sequencing of cDNA were performed as described elsewhere [27]. All reactions were performed in triplicate.

## 3 Results

### 3.1 Clinical details of patients

All five affected individuals (V:2, V:4, V:5, V:7, and V:8) were born to consanguineous parents and showed the onset of clinical manifestations between the ages of 30 and 38 years. Progressive choreoathetosis, orofacial dyskinesia, seizures, aggressive behavior, anxiety, and self-mutilation were common clinical features in all affected individuals. Non-coordinated mandibular movements and uncontrolled frequent lip biting were also observed (Table 1). Dysphagia and dysarthria were observed in only one affected individual (V:4). A history of drug exposure that would have caused the symptoms of extrapyramidal dysfunction was ruled out. Head drops, tongue-lip biting, slurred speech with vague words, and mild drooling were also observed in some affected individuals (V:2, V:4, and V:7). All the affected individuals were negative for the Kayser-Fleischer (K-F) ring in both corneas. The Montreal Cognitive Assessment (MoCA) was used to evaluate cognitive function. The scores were between 27 and 28 for most of the affected individuals.

**Table 1:** Clinical characteristics of four affected individuals with homozygous *VPS13A* synonymous variant.

Clinical Details/Demographics	V:2	V:4	V:5	V:7
Current Age	48	44	33	39
Age of onset	35	34	30	38
*Face*				
- Orofacial dyskinesia	No	Yes	Yes	Yes
*Neck*				
- Neck flexion, intermittent	No	Yes	No	No
*Gastrointestinal*				
- Dysphagia	No	Yes	No	No
- Drooling	No	Yes	No	No
*Feet*				
- Pes cavus	No	No	No	No
*Muscles, Soft Tissues*				
- Limb muscular atrophy	Yes	Yes	No	Yes
- Limb muscle weakness	Yes	Yes	No	Yes
*Central Nervous System*				
- Progressive choreoathetosis	N/A	Yes	Yes	N/A
- Orofacial dyskinesia	No	Yes	No	No
- Hyporeflexia	No	No	No	No
- Dysarthria	Yes	Yes	No	Yes
- Seizures	Yes	Yes	Yes	Yes
- Tics	No	No	No	No
- Dystonia	No	No	No	No
- Parkinsonism	No	No	No	No
- Caudate atrophy	NA	Yes	Yes	NA
- Putamen atrophy	NA	Yes	Yes	NA
- Dementia	Yes	No	No	Yes
*Peripheral Nervous System*				
- Hyporeflexia	No	No	No	No
- Areflexia	No	No	No	No
*Behavioral Psychiatric Manifestations*				
- Personality changes	Yes	Yes	No	Yes
- Mood changes	Yes	Yes	No	Yes
- Anxiety	Yes	Yes	Yes	Yes
- Disinhibition	Yes	No	No	No
- Psychosis	Yes	No	No	No
- Aggressiveness	Yes	Yes	Yes	Yes
- Self-mutilation of tongue and lips due to involuntary movements	Yes	Yes	Yes	Yes
*Haematology*				
- Acanthocytes	Yes	Yes	No	No
*Laboratory abnormalities*				
- Increased creatine kinase	No	No	No	No
- Normal serum lipoprotein levels	Yes	Yes	Yes	Yes

Blood and urine examinations were carried out to identify the functions of the liver, kidneys, and immune system. Table 2, Table 3 show the results of immunology, haematology, and clinical biochemistry examinations of the patient's (V:4) blood sample. The erythrocyte sedimentation rate (ESR), coagulation function, including partial thromboplastin time (PTT) and prothrombin time (PT), glycosylated hemoglobin, and autoantibodies, were normal. Echocardiography (ECG) and ultrasonography (USG) of the abdomen and radiographs of the upper and lower body parts were normal.

**Table 2:** Immunological, serological and haematological profile of an affected individual (V:5) of a family with homozygous synonymous variant in *VPS13A* gene.

Test	Result	Reference Range
**IMMUNOLOGY and SEROLOGY**		
Anti-Streptolysin O-Quantitative (ASOT)	138.30 U/ml	Upto 200 U/ml
Anti-Neutrophil cytoplasmic antibodies (cANCA)	2.3 U/ml	<5.0 U/ml
Anti-Neutrophil cytoplasmic antibodies (pANCA)	1.2 U/ml	<5.0 U/ml
Anti DNA – Double strand (A-DNA ds)	<1:10	<1:10
Anti DNA – Single strand (A-DNA ss)	<1:10	<1:10
Anti-Thyroglobin antibodies (A-TG)	10.00	Threshold - 115
Anti-Phospholipids antibodies (IgG)	2.0 U/ml	Negative: <10.0
Anti-Phospholipids antibodies (IgM)	1.0 U/ml	Negative: <10.0
**HAEMATOLOGY**		
Erythrocytes Sedimentation Rate (ESR)	21 mm/1hr	Up to: 25
WBC	8.24 × 10^3^/ul	4–11
Neutrophils Count	4.93 × 10^3^/ul	2–6.45
Lymphocytes Count	2.71 × 10^3^/ul	0.95–3.07
Monocytes Count	0.42 × 10^3^/ul	0.2–0.8
Eosinophils Count	0.16 × 10^3^/ul	0–0.4
Basophils Count	0.02 × 10^3^/ul	0–0.1
RBC	4.54 × 10^6^/ul	4–5.3
Haemoglobin (Hb)	13.00 g/dl	11.9–16
Haematocrit (PCV)	38.50 %	35–47
MCV	84.90 fL	80–96
MCH	28.60 pg/cell	26–33
MCHC	33.70 g/dl	30–37
RDW	13.10 %	11.5–15.5
Platelets	573 × 10^3^/ul	145–450

**Table 3:** Clinical biochemical profile of an affected individual (V:5) of a family with homozygous synonymous variant in *VPS13A* gene.

Test	Result	Reference Range
Fasting Blood Glucose (FBS)	107 mg/dl	55–110
Cholesterol - Total	130 mg/dl	No risk: <200
Cholesterol - HDL	59 mg/dl	No risk: >65
Cholesterol - LDL	66 mg/dl	Optimal: <100
Total Cholesterol/HDL Ratio	2.20	<4.5
Triglycerides (Trig.)	45 mg/dl	Normal: <15
Alanine Aminotransferase (ALT, GPT)	22 U/L	0–45
Aspartate Aminotransferase (AST, GOT)	31 U/L	0–43
Alkaline Phosphatase (ALP)	79 U/L	35–104
Bilirubin - Total	0.84 mg/dl	Up to: 1.2
Protein - Total	6.2 g/dl	6.6–8.7
Albumin	4.04 g/dl	3.5–5.2
Globulin	2.16 g/dl	2.1–3.7
Blood Urea Nitrogen (BUN)	15.9 mg/dl	6–21
Creatinine	0.61 mg/dl	0.50–0.90
BUN/Creatinine ratio	26.1	6.0–22.0
Uric acid	3.69 mg/dl	2.4–5.7
Iron	63 μg/dl	37–145
Calcium (Ca)	9.65 mg/dl	8.60–10.00
Thyroid stimulating hormone (TSH, Thyrotropin)	1.33 ulU/ml	027–4.2
25-Hydroxyvitamin D- Total (D2 + D3)	14.3 ng/ml	Deficient: <20

**Fig. 2:**
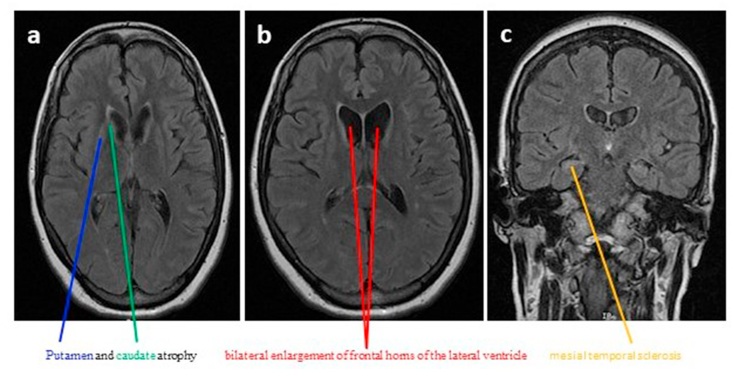
Brain MRI of individual V:5. Axial T2 FLAIR showing bilateral symmetrical caudate and putamen atrophy (a), AXIAL T2 FLAIR showing bilateral enlargement of frontal horns of the lateral ventricle (b), Coronal T2 FLAIR shows hyper-intense signals in both hippocampal regions suggestive of mesial temporal sclerosis (c).

Magnetic resonance imaging (MRI) and magnetic resonance angiography (MRA) of the brain were performed on two individuals (V:4 and V:5). MRI and MRA of the individual (V:4) were performed before disease onset. The Bicaudate index of this individual is 0.12. A normal brain structure was observed in this individual (V:4). Briefly, the brain parenchyma showed no areas of altered signal intensity or blood products. The ventricular system, brain stem, and cerebral hemisphere appeared to be normal. No midline shifts were observed. No extra- or intra-axial blood collection was observed, and the flow pattern of arteries sharing the circle of Willis was normal. Moreover, the carotid and vertebral basilar systems had no defects. Overall, the MRI and MRA of the brain of this patient (V:4) were unremarkable. A brain MRI of the individual (V:5) after disease onset showed enlargement of the frontal horns on both sides. A box-like configuration and mild generalized cerebral cortical atrophic changes were also observed. Moreover, T2 hyper-intense signals with some volume loss of both the putamen and bilateral symmetrical caudate head atrophy were observed. Based on these findings, this individual (V:5) was diagnosed with Huntington's disease (HD) or chorea HD. A few scattered minimal T2 FLAIR hyper-intense white matter foci were seen bilaterally. These are termed non-specific white matter spots. Moreover, minimal FLAIR hyper-intense signals were observed in both hippocampal regions. These were suggestive of mesial temporal sclerosis [Fig. 2a-c]. However, no brain parenchymal infarction, no focal lesions, no space-occupying lesions or any brain parenchymal herniation, no hydrocephalus, no sign of increased or decreased pressure, and no intracranial hemorrhage were seen. The sellar, parasellar, and suprasellar structures were unremarkable. Temporal bones, PNS, both orbits, and posterior fossa structures, including the brainstem, appear unremarkable. In conclusion, the MRI findings of this individual (V:5) were highly compatible with HD and mesial temporal sclerosis.

### 3.2 WES identified a synonymous variant in the *VPS13A* gene

The clinical presentation and availability of several affected individuals in the family led us to conclude that genetic defects are the underlying cause of the disease in this family. Therefore, the genomic DNA from the two affected individuals were subjected to WES. Sequencing reads were aligned, and variants were called and annotated. CAG repeats were excluded. Extensive filtration and prioritization identified several interesting variants in eight different genes (Table 4). Variants in the *POF1B* and *RPS6KC1* genes were not considered further due to the benign nature of the variants. Variants in *JAG1, TTN,* and *DSTYK* were found with an allele frequency of ≥5 % in the in-house exome database of healthy individuals from the same ethnicity. Deleterious variants in *FKRP* and *RSAD2* were not segregating with the phenotype. Therefore, a high-quality shared variant (c.5040C > T) in *VPS13A* gene was considered the most promising candidate variant for the disease in this family. This variant is not changing (p.G1680G) amino acid at position 1680 in the protein. However, this variant is highly conserved, rare, and absent in gnomAD. Moreover, the CADD score for this variant was high.

**Table 4:** Rare variants identified in exome sequence data of the two affected individuals.

Gene Name	Variant	Amino Acid Change	Zygosity	Aggregated Prediction
*FKRP*	c.845G > C	p.Gly282Ala	Homozygous	Deleterious
*JAG1*	c.2666G > A	p.Arg889Gln	Heterozygous	Uncertain
*TTN*	C.39566_39568delAAG	p.Glu13189del	Heterozygous	Not available
*POF1B*	c.986G > A	p.Arg329Gln	Hemizygous	Benign
*VPS13A*	c.5157C > T	p.Gly1719Gly	Homozygous	Not available
*RSAD2*	c.575G > T	p.Gly192Val	Heterozygous	Deleterious
*DSTYK*	c.635T > C	p.Met212Thr	Homozygous	Uncertain
*RPS6KC1*	c.2719G > A	p.Gly904Ser	Homozygous	Benign

### 3.3 Variant (c.5040C > T) segregates in a family in an autosomal recessive manner

**Fig. 3:**
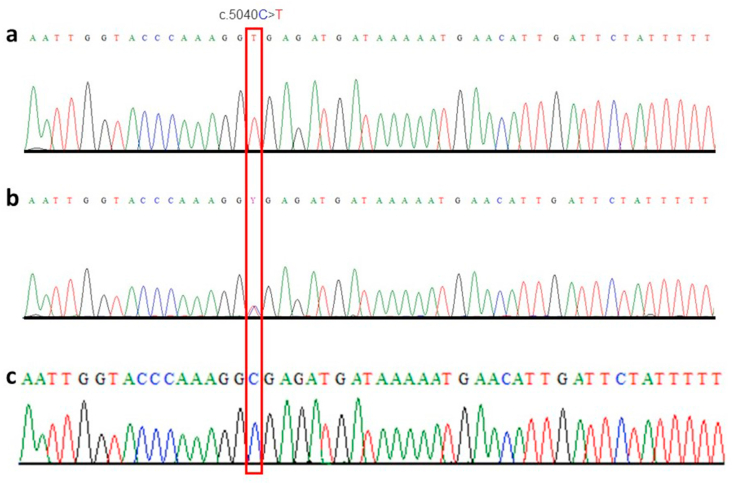
Partial sequence of exon 41 of the *VPS13A* gene. Position of variant (c.5040C > T) is highlighted in a red rectangle. Homozygous mutant sequence chromatogram of affected individuals (a) and heterozygous sequence (b) of both parents and two unaffected individuals (V:3 and V:6). Unaffected individual V:1 and control individuals from the same population shows wild type sequence (c).

Primer pairs flanking the variant site were used to amplify the partial sequence of exon 41 of *VPS13A* in all available members of the family. The amplicons were purified and amplified with sequencing primers using dideoxy chain termination chemistry. The reads were sequenced using an ABI3500 instrument. The sequence of each individual was compared to the reference sequence to identify homozygotes and heterozygotes. All patients were found to be homozygous for the variant (c.5040C > T) (Fig. 3a), and both parents were heterozygous (Fig. 3b) and unaffected siblings were either heterozygous or wild-type (Fig. 3c).

### 3.4 Bioinformatics analysis predicted that the variant generated a new splice site

The novel variant c.5040C > T (p.G1680G), found in this study, is synonymous; though, it was not present in the gnomAD, HGMD, 1000 Genomes, and ExAC databases. The variant is positioned in the middle of exon 41 of 72 exons of the *VPS13A* gene and was consistently predicted to create a new splice donor site by various online tools, including the Alternative Splice Site Predictor (ASSP) tool (http://wangcomputing.com/assp/index.html), Spliceator (http://www.lbgi.fr/spliceator/), and NetGene2 (https://services.healthtech.dtu.dk/service.php?NetGene2-2.42) (Table 5). Given the reliable *in silico* prediction of a cryptic splice-site due to the synonymous variant and the absence of a homozygous variant in a large population sequencing databases, homozygous nature of variant in all patients of a family, this variant was taken as likely pathogenic according to the ACMG guidelines (PM2_supporing + PP3 + PM3_strong + PP1). Hence, we can confidently considered this variant as an underlying cause of autosomal recessive CHAC.

**Table 5:** The variant was consistently predicted to be a novel cryptic splice donor site by various splice site algorithms.

Tool	Splicing cite	Score/Confidence	Sequence
Spliceator	Donor	0.994	AAAG**GT**GAGA
NetGene2	Donor	0.93	GTACCCAAAG^**GT**GAGATGAT

### 3.5 The reverse transcription PCR confirmed the generation of a new splice site

The variant (c.5040C > T) found in this study is positioned in the middle of exon 41 of the *VPS13A* gene. *In silico* tools indicated a constraint for cytosine at position 5040. Notably, thymine substitutions create a GT motif that is predicted to act as a cryptic alternative splice donor (Table 5). The effect of this variant was evaluated by blood-derived RNA from patients (homozygote for the variant c.5040C > T) and unaffected (heterozygotes for the variant) individuals. Reverse transcription polymerase chain reaction (RT‐PCR) was performed with primers targeting exons 39 and 42. Product was sequenced using Sanger approach. In both heterozygous and homozygous individuals, sequencing confirmed the predicted alternative splicing event joining mid‐exon 41 to exon 42. In conclusion, the RNA sequence of homozygous individuals is compatible with the presence of alternatively spliced, out-of-phase mRNA, leading to RNA deletion and premature termination.

## 4 Discussion

CHAC is a rare autosomal recessive neuromuscular disorder characterized by choreiform movement and acanthocytosis of RBCs. It is an adult-onset disease, and its clinical spectrum is variable and wide [28,29]. Speculated RBCs can be used as a diagnostic hallmark; however, these features are occasionally absent or may appear late. High levels of creatine kinase (CK) and liver enzymes (ALT, ASP, and ALP) are also common laboratory findings in CHAC patients.

In the present study, we identified the underlying cause of neurological disorder in a large five-generation consanguineous family with multiple affected individuals. These individuals were initially misdiagnosed as having Huntington's disease (HD) or mesial temporal sclerosis. CHAC and HD are neurodegenerative conditions that share clinical and neuropathological features [9]. Sequencing of the complete coding region of the genome detected a rare synonymous variant (c.5040C > T) in the *VPS13A* gene in this family. The *VPS13A* gene has the highest expression in the pons, hippocampus, and cerebellum of the brain [30]. Therefore, patients with VPS13A gene mutation show abnormalities in caudate, putamen and hippocampal regions. Thus, this genetic study helped us reach a correct diagnosis of chorea in our patients. Moreover, this study highlights the importance of rare, potentially damaging coding synonymous variants in disease pathogenesis and emphasizes the need to consider synonymous variants during the analysis of large genomic variant data. CHAC show progressive neurological symptoms, therefore, an intrafamilial phenotypic variability was observed in this family.

The variant (c.5040C > T) detected in this study is in the coding part of exon 41 of the *VPS13A* gene. Although this variant does not change any amino acid in the protein (p.G1680G), it was reliably predicted to generate a novel splice donor site using multiple splice-site prediction algorithms, including ASSP, Spliceator, and NetGene2 tools. This variant is predicted to cause abnormal splicing of pre-mRNA. Based on the *in silico* prediction of a new splice-site, absence of variant in population databases, and presence of the variant in homozygous state in all patients, and the segregation of variant with the clinical features in the large family, this variant was considered as likely pathogenic according to the American College of Medical Genetics (ACMG) guidelines. Therefore, our data supports the diagnosis of autosomal recessive chorea-acanthocytosis due to VPS13A deficiency. These findings are important for genetic screening of at risk families for detection of homozygous individuals at the early stages. Moreover, the variant can be incorporated in prenatal screening program for the identification of at-risk pregnancies. The earlier detection of inherited diseases permits various interventions and decisions for better management of pregnancy. Prenatal therapeutic approaches can be used by inactivating, activating or alteration of the mutant gene [31]. CRISPR/Cas9 show great potential for the management of genetic defects at the level of foetus [32].

## 5 Conclusion

In summary, we report the phenotypic manifestations and molecular findings in a Saudi family with a homozygous synonymous variant in the *VPS13A* gene. We also characterized the mechanisms underlying the pathogenesis of the cryptic exonic splicing variant (p.Gly1680Gly). Furthermore, we found that this variant affected RNA splicing by generating a cryptic splice site.

## CRediT authorship contribution statement

**Majed Alluqmani:** Methodology, Investigation, Funding acquisition, Data curation. **Shahid Iqbal:** Visualization, Resources, Methodology, Investigation, Data curation. **Sulman Basit:** Writing – review & editing, Writing – original draft, Validation, Supervision, Software, Project administration, Investigation, Conceptualization.

## Ethics statement

The research ethics committee (REC) of college of medicine, Taibah University, Medina, provided ethical approval for this study. The study approval number is 036-1441.

## Data availability

The fastq files of a patient containing whole exome sequencing reads have been submitted to the sequence reads archive (SRA) of the NCBI. The submission ID is SUB14773274.

## Consent for the publication of clinical and genetic data

Informed written consents were obtained from all the participants to publish clinical and genetic data.

## Funding

The authors extend their appreciation to the Deputyship for Research & Innovation, 10.13039/501100011821Ministry of Education in Saudi Arabia for funding this research work through project number 442/70. Also, the authors would like to extend their appreciation to Taibah University for its supervision support.

## Declaration of competing interest

The authors declare that they have no known competing financial interests or personal relationships that could have appeared to influence the work reported in this paper.
